# Dynamics and diversity of bacteria associated with the disease vectors *Aedes aegypti* and *Aedes albopictus*

**DOI:** 10.1038/s41598-019-48414-8

**Published:** 2019-08-21

**Authors:** Kelly L. Bennett, Carmelo Gómez-Martínez, Yamileth Chin, Kristin Saltonstall, W. Owen McMillan, Jose R. Rovira, Jose R. Loaiza

**Affiliations:** 1 Smithsonian Tropical Research Institute, Apartado 0843-03092, Balboa, Ancon Panama; 2 Instituto de Investigaciones Científicas y Servicios de Alta Tecnología, Ciudad del Saber, Apartado, 0843-01103 Ciudad de Panamá Panama; 3 Programa Centroamericano de Maestría en Entomología, Universidad de Panamá, Ciudad de Panamá, Panama

**Keywords:** Metagenomics, Metagenomics, Microbiome

## Abstract

*Aedes aegypti* and *Aedes albopictus* develop in the same aquatic sites where they encounter microorganisms that influence their life history and capacity to transmit human arboviruses. Some bacteria such as *Wolbachia* are currently being considered for the control of Dengue, Chikungunya and Zika. Yet little is known about the dynamics and diversity of *Aedes*-associated bacteria, including larval habitat features that shape their tempo-spatial distribution. We applied large-scale 16S rRNA amplicon sequencing to 960 adults and larvae of both *Ae*. *aegypti* and *Ae*. *albopictus* mosquitoes from 59 sampling sites widely distributed across nine provinces of Panama. We find both species share a limited, yet highly variable core microbiota, reflecting high stochasticity within their oviposition habitats. Despite sharing a large proportion of microbiota, *Ae*. *aegypti* harbours higher bacterial diversity than *Ae*. *albopictus*, primarily due to rarer bacterial groups at the larval stage. We find significant differences between the bacterial communities of larvae and adult mosquitoes, and among samples from metal and ceramic containers. However, we find little support for geography, water temperature and pH as predictors of bacterial associates. We report a low incidence of natural *Wolbachia* infection for both *Aedes* and its geographical distribution. This baseline information provides a foundation for studies on the functions and interactions of *Aedes*-associated bacteria with consequences for bio-control within Panama.

## Introduction

The arboviral disease vectors of Dengue (DENV) and chikungunya (CHIKV) viruses, *Aedes aegypti* and *Aedes albopictus* are invasive mosquitoes that utilise the same habitats and hosts as they expand and naturalise. This includes the use of water-filled containers around human settlements for immature development, where larvae compete for space and resources, feeding on microorganisms or detritus in the water. However, little descriptive information exists to date about whether *Aedes* species exhibit niche partitioning in the microorganisms they encounter and utilise. Furthermore, since mosquitoes acquire a large proportion of their bacterial microbiota as larvae, resource use in aquatic habitats are likely to impact the core microbiota of adult mosquitoes^1,2^. This is important since the capability of a female mosquito to transmit pathogens to humans (e.g., vectorial capacity) is directly influenced by the microbiota, through the production of metabolites^3^, resource competition^4^, regulation of miRNA’s^5,6^ and alteration of the insect immune response^7^. Yet, very little is known currently as to how the larval microbial community influences the microbiota of adult mosquitoes. Resident microbiota can also alter mosquito life history traits important for ecological success and disease transmission, impacting on host fitness through nutrient acquisition^8^, reproduction^9^, development^1,10,11^ and predator and pathogen defence^12,13^. Hence, microbial communities could influence the outcome of inter-specific competition at the larval stage and the vectorial capacity of adult *Aedes* mosquitoes, ultimately leading to changes in human risk of exposure to arboviral diseases such as Dengue, Chikungunya and Zika. Decoding features of the larval habitat that shape *Aedes*-bacterial interactions and understanding the tempo-spatial dynamics of *Aedes* microbes, including within and between-species differences, provides the foundation on which to unravel their epidemiological impact.

Because mosquito-associated bacteria could alter disease transmission, there is interest in using intrinsic bacteria to modify vector populations. This provides an alternative to chemical spray, which is compromised by the widespread development of insecticide resistance. The intracellular bacterium *Wolbachia* has been proposed as a strategy to diminish disease transmission through the infection of *Aedes* mosquitoes. *Wolbachia* can inhibit the replication of Yellow Fever (YFV), Dengue, Chikungunya and Zika viruses both *in vitro*^14–22^ and *in vivo*^23^. Moreover, *Wolbachia* has been proposed as a means to control mosquito populations through the sterility induced by cytoplasmic incompatibility. The strain of *Wolbachia* currently proposed for mosquito control, wMelPop, successfully spreads through the population because of a fitness advantage conferred by the infected female. This occurs because males infected with the wMelPop strain cannot produce viable eggs on reproduction with un-infected females while infected females are able to produce eggs that hatch with both infected and uninfected males^24^. The effective spread of introduced *Wolbachia* among natural populations relies on a gene drive system that can be impacted by the interaction of different *Wolbachia* strains and bacterial community members, producing variable fitness consequences^25,26^. Therefore, establishing the natural occurrence and geographic distribution of *Wolbachia* is central to the design and implementation of vector/disease mitigation strategies. Although commonly found in wild *Ae*. *albopictus*^27^, natural infection with *Wolbachia* in *Ae*. *aegypti* has only been recently described^1^, which could be perhaps due to a lack of studies specifically targeting *Wolbachia* in wild populations of *Ae*. *aegypti* at a global scale.

Herein, we conduct extensive collections of *Aedes* mosquitoes across the entire country of Panama and use 16S rRNA metabarcoding to characterise their microbiota. To date, efforts to characterise the bacterial community of wild mosquitoes have been limited to over a small geographic scale^1,2,25,28–33^. This includes very little related work on the arboviral disease vectors *Ae*. *aegypti* and *Ae*. *albopictus*, despite their considerable impact on public health^34–36^. In Panama, the two species have different population histories; *Ae*. *aegypti* has been present since the 17^th^ Century^34,37^, whereas *Ae*. *albopictus* was recently introduced into Panama in 2002. There is also evidence for repeated introductions into Panama of both species from the Americas and Europe^38^. Preliminary data on mosquito occurrence across Panama supports an on-going pattern of competitive displacement, whereby the invasive spread of *Ae*. *albopictus* has modified the species distribution of *Ae*. *aegypti*; species are rarely found in the same oviposition site throughout their geographic range while *Ae*. *aegypti* are more associated with populated areas.

Since the geographical distribution of *Aedes* species (e.g., co-existence) has not been fully characterised across Panama, our intention is not to test whether bacteria are shared across the same oviposition sites, but rather we use country-wide data i) to describe the intra- and inter-species microbial communities associated with larval and adult stages of *Ae*. *aegypti* and *Ae*. *albopictus*, and ii) to determine whether features of larval habitats including geographic location, type of container material and physical variables of the water (pH and temperature) influence the microbiota of these mosquitoes. Furthermore, given the interest in using *Wolbachia* as a biocontrol strategy to diminish arboviral disease in Panama, we use conventional PCR and Sanger sequencing on 16S *Wolbachia* positive samples to describe *Wolbachia* strain composition and geographic distribution across the country. Therefore, in addition to its ecological and epidemiological consequences, characterisation of the bacterial community of *Aedes* mosquitoes would provide valuable baseline information for trials of vector population control with genetically-engineered bacteria.

## Results

### Composition and structure of *Aedes*-associated bacterial communities

**Figure 1:**
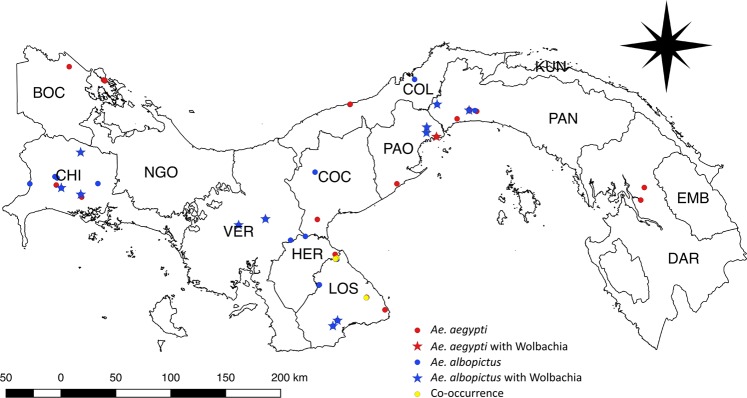
Sampling locations of *Aedes* mosquitoes across provinces and indigenous territories (also known as “*Comarcas*”) of Panama, including those confirmed to be infected with *Wolbachia*: Bocas del Toro (BOC), Chiriquí (CHI), Veraguas (VER), Herrera (HER), Los Santos (LOS), Coclé (COC), Colón (COL), Panamá Oeste (PAO), Panamá (PAN), Darién (DAR), Ngöbe Buglé (NGO), Kuna Yala (KUN), and Emberá (EMB).

**Figure 2:**
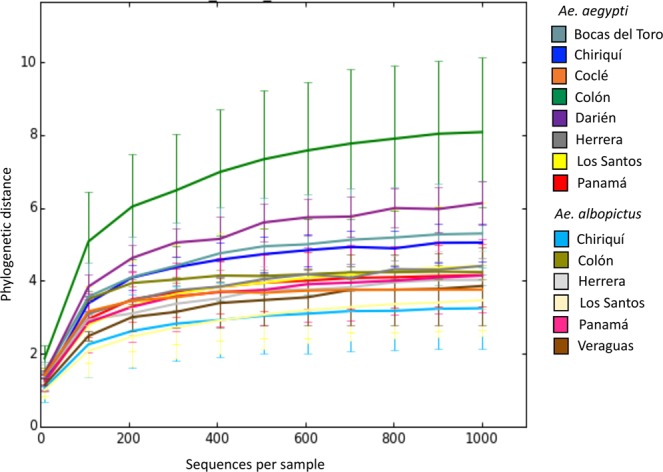
Alpha diversity of the bacterial communities found in larvae and adults of *Ae*. *aegypti* and *Ae*. *albopictus* collected from human inhabited environments across Panama as distinguished by the province from which they were collected. Mean Faith’s phylogenetic diversity (±SE) of each sample group is shown across different sequencing depths.

Preliminary sequencing of seven pools of six mosquitoes each captured a comparable level of bacterial diversity to individual component mosquitoes (Mann Whitney U of Faith’s Phylogenetic Diversity values, W = 58, P > 0.05), therefore informing our decision to process a larger number of individuals by pooling mosquitoes of the same species from the same oviposition site. In total, 4,921,352 sequence reads of bacterial 16S rRNA gene amplicons were generated from DNA pools and individually processed mosquitoes representing 960 samples of immature and adult *Ae*. *aegypti* and *Ae*. *albopictus*. This included 75 pools of *Ae*. *albopictus* representing 30 sampling sites, 79 pools of *Ae*. *aegypti* representing 30 sampling sites, 20 individual adult *Ae*. *albopictus* from seven sites and 16 individuals of adult *Ae*. *aegypti* from six sites. In total, we sampled 59 widely distributed natural habitats across Panama (Fig. 1, Table S1) and recorded co-existence of both *Aedes* at eight of the same oviposition sites in Panama, Los Santos and Chiriquí on the border with Costa Rica (~5%). The mean number of reads per pool or individual sample was 27,341 ± SE 3,310. Rarefaction curves were close to saturation at a sampling depth of 1,000, indicating that the bacterial diversity present in *Aedes* mosquitoes was captured (Figs 2, S1). After quality filtering, 3,568,301 sequences were retained from 681 samples, including 58 pools (26% larvae) and 10 individual adult *Ae*. *aegypti* and 51 pools (29% larvae) and 17 individual adult *Ae*. *albopictus*. These samples comprised 721 unique operational taxonomic units (OTUs), averaged at 48 to 52 OTU’s per individual or mosquito pool.

**Figure 3:**
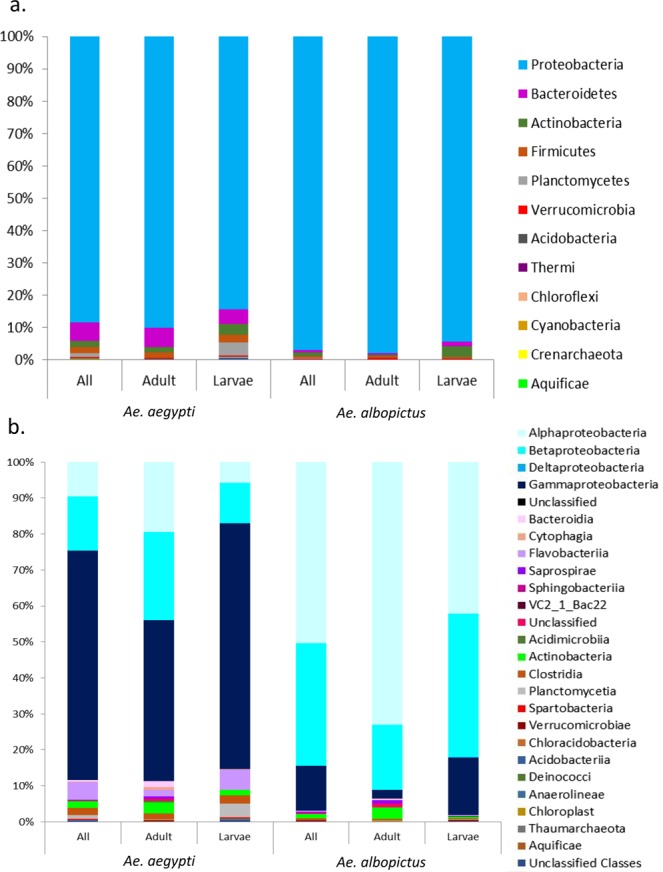
Bar plot of the relative abundances of (**a**) the bacterial phyla and (**b**) the bacterial classes within *Ae*. *aegypti* and *Ae*. *albopictus* summarized across all samples and compared to the bacterial profiles of the adults and larvae for each species.

In total, the bacterial communities analysed composed 12 phyla, 24 classes, 51 orders, 76 families and 115 genera (Table S2). Only 16 genera had a relative abundance over 1%, suggesting that few bacterial groups are able to colonise the mosquito (Table S2). We found that members of *Proteobacteria* dominated the communities, composing upwards of 88% of the identified OTU’s for each of the two mosquito species, while *Actinobacteria*, *Bacteroidetes* and *Firmicutes* were also represented although at lower relative abundances. All other Phyla had an overall relative abundance of less than 2% (Fig. 3, Table S3).

### *Aedes* intra- and inter-species bacterial diversity

**Figure 4:**
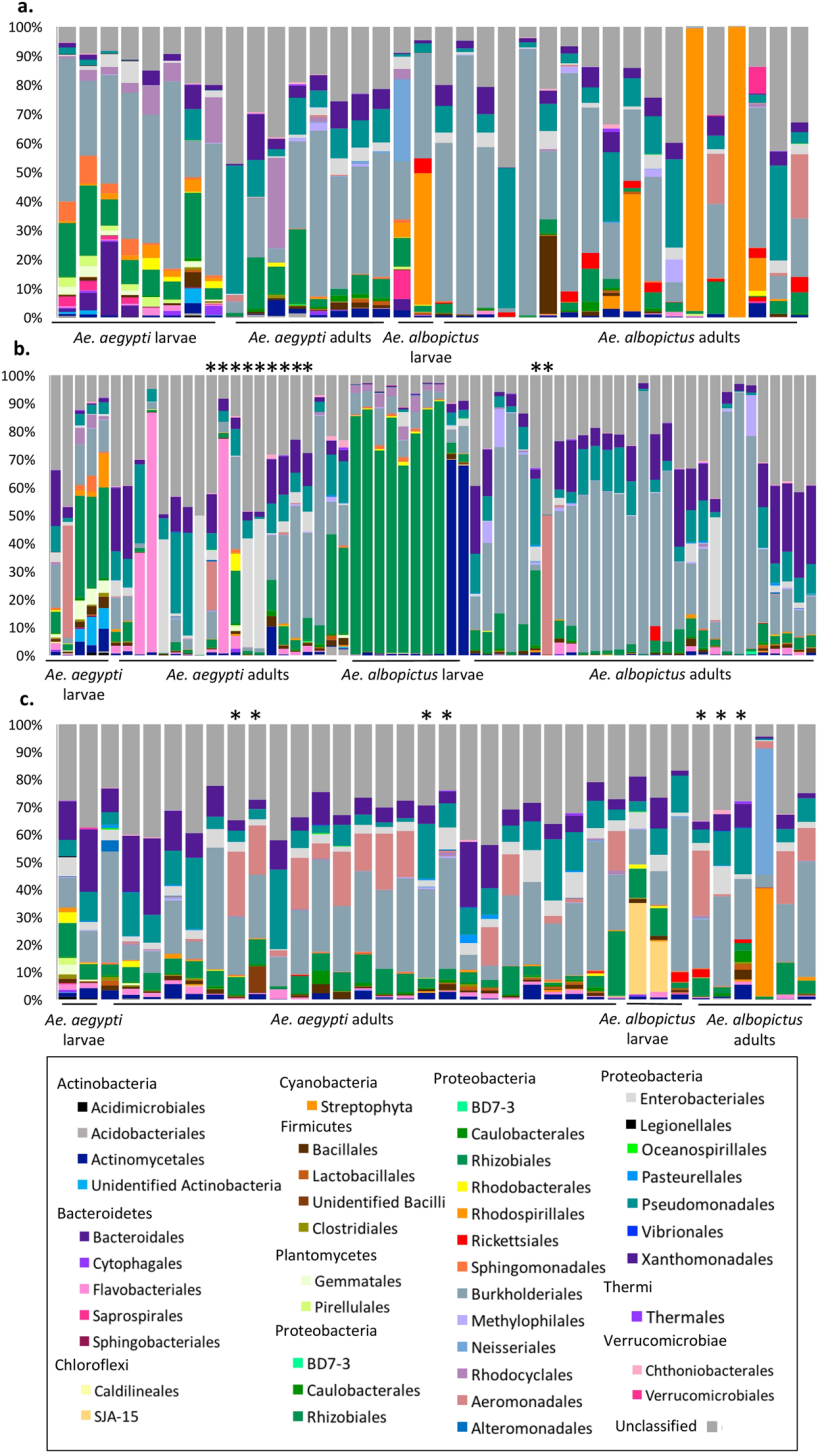
Bar plot to show the proportion of different bacterial classes within each mosquito or pool of mosquitoes tested from. (**a**) Western Panama including Bocas del Toro, Chiriquí, Herrera and Veraguas. (**b**) Los Santos and (**c**). Eastern Panama including Colón, Coclé, Darién and the province of Panama. Bacterial classes with an overall relative abundance of at least 1% within either species are specified. * Indicate an inter-species comparison of equivalent sampling sites.

We found a high degree of intraspecific variation in the bacterial community of both *Aedes* species, demonstrated by high average Bray-Curtis distances of 0.80 and 0.84 for *Ae*. *aegypti* and *Ae*. *albopictus*, respectively. In support of this, certain bacteria members were prevalent in one pool of individuals but rare or entirely absent from another, with the highest variation between members of *Flavobacteriales*, *Rhodocyclales*, *Rhodospirillales* and *Aeromonadales* (Fig. 4). For instance, *Flavobacteriales* (*Bacteroidetes*) and *Rhodospirillales* (*Proteobacteria*) although frequently found in smaller proportions, dominated three pools of adult *Ae*. *aegypti* from the Azuero Peninsula, and two pools of adult *Ae*. *albopictus* from Chiriquí, respectively, contributing between 52 and 90% of the bacterial OTU’s in these places. Furthermore, we observed a higher bacterial diversity in larvae with a significantly different microbial community than newly emerged adults in both *Ae*. *aegypti* (PERMANOVA of unweighted UNIFRAC distances, pseudo-F = 5.16, P < 0.01) and *Ae*. *albopictus* (PERMANOVA of unweighted UNIFRAC distances, pseudo-F = 3.35, P < 0.05) (Tables 1, S1, Fig. S1).

**Table 1:** Mean number of operational taxonomic units (OTU’s), Shannon’s Diversity values (Shannon’s D), Faith Phylogenetic diversity (Faith PD) and Evenness Index for each *Aedes* species in Panama.

Species	OTU’s	SE ±	Shannon’s D	SE ±	Faith PD	SE ±	Evenness	SE ±
*Ae*. *aegypti*	60.50	4.03	4.43	0.13	4.79	0.21	0.77	0.02
Adults	54.08	3.94	4.31	0.15	4.31	0.18	0.77	0.02
Larvae	85.40	10.07	4.88	0.28	6.68	0.47	0.77	0.03
*Ae*. *albopictus*	39.25	2.00	3.57	0.13	3.51	0.13	0.69	0.02
Adults	37.55	2.29	3.68	0.16	3.52	0.14	0.72	0.02
Larvae	47.80	3.39	3.22	0.21	3.91	0.23	0.58	0.04

**Figure 5:**
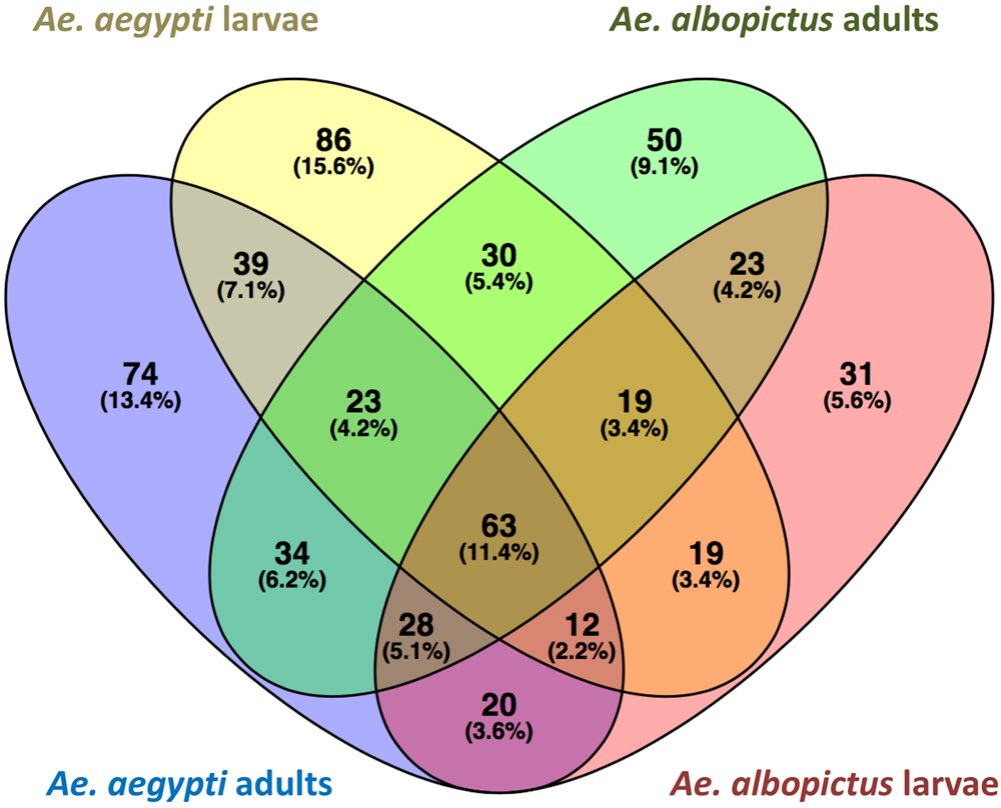
Venn diagram showing the numbers and proportions of OTU’s shared between the adults and larvae of *Ae*. *aegypti* and *Ae*. *albopictus*.

A large proportion of bacterial OTU’s (67.7%) were shared between samples of *Ae*. *aegypti* and *Ae*. *albopictus* overall, although a lesser extent was shared between species on comparison of either only adults or larvae (59.4% and 47.8% of OTU’s, respectively) (Fig. 5). Of the 8 of 59 oviposition sites where both species co-occurred, three out of eight (~38%) oviposition sites retained in the analysis after quality filtering, provided a one to one species comparison of adult mosquitoes and revealed the same trend of shared bacterial community members, yet high intraspecific variability (Fig. 4). Despite species sharing a considerable proportion of bacteria, we found several rare taxa unique to *Ae*. *aegypti*, reflected in a higher bacterial diversity within both adults (Mann-Whitney U of Faith’s PD, W = 1917, P < 0.01) and larvae (Mann-Whitney U of Faith’s PD, W = 216, P < 0.01) of this species when compared to *Ae*. *albopictus* (Tables 1, S4, Fig. S1). This trend holds within the three sites of co-existence, with a higher Faith’s phylogenetic distance for *Ae*. *aegypti* (4.05 ± 1.28) compared to *Ae*. *albopictus* (3.56 ± 1.51). Furthermore, random forest analysis can successfully assign adult *Ae*. *aegypti* and *Ae*. *albopictus* to the correct species class with a high accuracy of 0.82 for both although estimates for larvae were less exact, with predicted accuracies of 0.67 and 1.00, respectively. The fact that this accuracy is particularly lower for *Ae*. *aegypti* than for *Ae*. *albopictus* supports a higher degree of bacterial variability at the immature stage than as emergent adults with Bray-Curtis distances of 0.79 and 0.76, for this species respectively. Indicator species analysis did not identify any OTU’s characteristic of *Ae*. *aegypti* or *Ae*. *albopictus* after Benjamini & Hochberg correction for multiple tests (Tables S5 and S6).

### Larval habitat features and bacterial composition

**Figure 6:**
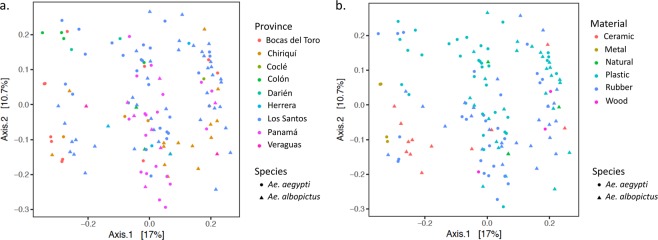
PCoA plots of UNIFRAC distances of the microbial community of *Ae*. *aegypti* and *Ae*. *albopictus* taken from (**a**) different provinces of Panama and (**b**) oviposition site materials.

We observed few pertinent differences between both the bacterial community of larvae and adult *Aedes* species due to larval habitats features including geographic distribution, type of container material and associated environmental variables of the water. Principle Coordinates Analysis (PCoA) revealed no clear differences between the bacterial communities for the majority of compared categories, with only few statistically significant comparisons based on PERMANOVA between oviposition sites having different container materials and geographic distribution (Tables S7, S8, Fig. 6). For instance, PCoA revealed that mosquito species developing in containers with metal and ceramic tend to harbour a distinct community of microbes. Indeed, a greater proportion of *Bacteroidia* and *Saprospirae* were isolated from *Ae*. *aegypti* developing in metal containers while a greater proportion of *Alphaproteobacteria*, namely *Rhizobiales*, *Sphingomonadales*, *Rhodocyclales* and *Rhodobacterales*, were isolated from *Ae*. *albopictus* developing in ceramic (Fig. 6).

Water temperature and pH of the larval habitat had no significant effect on the microbiota acquired by the mosquito host (Fig. S3).

### *Wolbachia* occurrence and distribution

We found *Wolbachia* 16S rRNA positive samples in both *Aedes* species. This included 11 pools and five individuals of *Ae*. *albopictus* (~15% samples), of both adults and larvae originating from plastic and ceramic containers and used-tyres widespread across Panama. *Wolbachia* 16S rRNA was also amplified and sequenced from one adult of *Ae*. *aegypti* originating from a plastic container in Veracruz, Panama (Fig. 1). Amplification of the *wsp* gene in 11 samples of *Ae*. *albopictus* from the provinces of Panama, Los Santos and Chiriquí revealed that all tested individuals were infected with the same strain of *Wolbachia* (wAlB) within supergroup B, known to occur naturally and widely in *Ae*. *albopictus* (Strain id 1847 included within the https://pubmlst.org database). The *wsp* gene could not be amplified from *Ae*. *aegypti*, for which *Wolbachia* was detected at a very low relative abundance (0.003%) compared to *Ae*. *albopictus* (0.4% overall or between 0.01–0.8% per *wsp* positive sample).

## Discussion

### Dynamics and diversity of *Aedes*-associated bacteria

Here, we provide the first descriptive study of the bacterial community within the arboviral disease vectors, *Ae*. *aegypti* and *Ae*. *albopictus* in Panama. We reveal that the core microbiota of both *Aedes* species is underpinned by extensive intraspecific variation including notable differences between conspecific mosquitoes collected from the same geographical location and comparable oviposition sites. This outcome suggests that individuals of both species acquire the basis of their microbiota during larval development although many OTU’s are either rare or variable in space and time, thus reflecting a high degree of stochasticity in their natural environment. The core microbiota of *Aedes* species was dominated by few bacterial phyla, which is similar to that previously described for *Anopheles*, *Culex* and *Aedes* mosquitoes^25,29,31,39–43^. Furthermore, in agreement with previous studies, we found that bacterial classes associated with *Aedes* mosquitoes included gram-negative *Gammaproteobacteria*, *Betaproteobacteria*, *Alphaproteobacteria*, *Bacteroidia* and *Flavobacteria* and gram-positive *Actinobacteria*, suggesting that these microbes have either evolved a close relationship or are the more prolific bacterial types within the mosquito host^25,44^.

We found differences between the bacterial composition of larvae and adults in both *Aedes* species, with a higher taxonomic diversity present in the former life stage. This is expected given that along with permanent bacteria, transient bacteria acquired for nutrition will also be detected in larvae, if they remain undigested in the gut, and because adult mosquitoes lose bacterial diversity as they shed their skin on pupal emergence^45^. Since we pooled whole larvae, we cannot determine whether the OTU’s differentially abundant between the life stages are gut microbes. However, findings indicate that *Ae*. *aegypti* and *Ae*. *albopictus* share a fairly similar niche in terms of the bacterial community they host at the larval and adult stages, except for the presence of some rare and unique bacterial community members in *Ae*. *aegypti*. That *Ae*. *aegypti* has a higher bacterial diversity than *Ae*. *albopictus* at the larvae and adult stages suggests it could be more a generalist aquatic feeder or has a higher tolerance of bacterial commensalism for which its members may have evolved specific functions^44^. However, that these mosquitoes tend to share a large proportion of bacterial types signals the need for further work to understand whether resource competition in association with bacterial acquisition can impact on mosquito development and survival.

Variation in the microbiota of mosquitoes at the intra- and interspecific levels has been previously linked to environmental variation in container breeding sites, with three mosquito species showing a more similar microbial profile at nearby versus more distant sites in the south-eastern United States and local clustering of meta-bacterial profiles of *Anopheles* in Burkina Faso^1,28^. However, in the case of invasive and ecologically similar *Aedes* species, the outcomes of high intraspecific and low interspecific bacterial variation cannot be easily explained by differences in larval habitat features, including geographic distribution, type of container material and associated environmental variables of the water. We observed few meaningful differences between categories of larval habitat features, with the only significant differences observed between bacterial communities of *Ae*. *aegypti* from metal containers and *Ae*. *albopictus* from ceramic containers. Moreover, we found no support for geography and water environmental variables as meaningful predictors of *Aedes* bacterial associates. However, it is likely that many small effects from biotic and abiotic factors act in combination to explain the core microbiota of *Aedes* mosquitoes, including a variety of unexplored factors such as bacterial competition in the host, interspecific competition in the aquatic environment, variation in the water and surrounding environment and genetic background^46–48^.

### The role of bacteria in shaping *Aedes* phenotype

The high variation in the microbial community among mosquito pools of the same species could translate to high variation in vector competence within *Ae*. *aegypti* and *Ae*. *albopictus*. Studies relating variation in the microbial community to the arboviral competence of *Aedes* mosquitoes has the potential to reveal bacterial members important in either mitigating or facilitating disease dissemination. Information on the intraspecific variability of vector competence of either *Aedes* is currently lacking within Panama. Yet, if the microbial community is a strong determinant of disease transmission ability and based on the high intrapopulation variability that we have observed, it is expected that vector competence would likewise be highly variable within a single population. Previous studies have demonstrated high intraspecific variation in vector competency in Brazilian populations of *Ae*. *aegypti*^49–51^, which could be mediated by intrinsic bacteria. For example, members of the genus *Serratia*, which we found to be 1% prevalent in *Ae*. *aegypti*, have been shown to increase DENV-2 susceptibility in the females of this species^52^.

The microbiota identified in both mosquito species should be the target of further experimental work in order to investigate their functions and role in mosquito fitness. Bacterial residents can contribute to the hosts fitness by influencing their development^1,10,11,53^, reproduction^54^ and nutrition^55,56^ with those present in a wide-range of taxa potentially involved in their basic functions^57^. Little is currently known about the functional roles and interactions of bacteria within mosquito hosts, but several microbes identified in this study have been previously implicated in blood and nectar assimilation (e.g. *Corynebacterium*, *Serratia*), known to fix nitrogen (e.g. *Xanthobacter*, *Novispirillum*), act as an attractant to gravid females (e.g. *Enterobacter*, *Acinetobacter)* or have the ability to impact on reproduction (e.g. *Stenotrophomonas)*^44,46,54,58–60^ (Table S2). Additionally, it has been found that the presence of a particular bacteria (eg. *Wolbachia*, *Chromobacteria*, *Proteus*, *Paenibacillus*) can inhibit or promote infection with viruses^61–63^. Exploration of the relationship between mosquito bacterial communities and intraspecific disease competence would be of value along with work targeting the ecological factors that underpin the relevant variability in their microbiota.

### Implications for *Wolbachia* bio-control in Panama

The presence of *Wolbachia* in *Aedes* mosquitoes has important ramifications for attempts at biocontrol that introduce either naturally occurring or modified *Wolbachia* strains to reduce the transmission of Dengue^17,20,64^, Chikungunya^14,17,18^ and Zika viruses^65^. So as to drive *Wolbachia* spread through a natural mosquito population on the basis of cytoplasmic incompatibility, there must be sufficient infected males present to confer a fitness advantage to the infected female^66^. The wALBb strain we have observed has the potential to facilitate arboviral control and may indeed prove a more acceptable disease control strategy than other genetically modified *Wolbachia* strains, given its natural occurrence within Panama. Infection with wALBb is known to reduce DENV replication within *Ae*. *aegypti*^67^, although further investigation is needed to understand its impact on viral replication within *Ae*. *albopictus*, including its implications for other arboviruses and population specific effects to be considered for biocontrol. We have observed widespread natural infection of *Wolbachia* wALBb within *Ae*. *albopictus* across Panama, although this is not as common as in previous studies, which report infection rates of over 90% in populations sampled from USA^29^, Malaysia^27^, Thailand^68^ and La Reunion^69^. Disease control using wALBb induced cytoplasmic incompatibility would therefore require the release of a large number of laboratory infected mosquitoes in order to push the infection rate over the required threshold. The presence of naturally occurring *Wolbachia* has the potential to interfere with cytoplasmic compatibility induced by introduced *Wolbachia* strains, or alternatively alter fitness effects and so the dynamics of the proposed gene drive system^26^. However, if the release of the proposed strain for population control, wMelPop, is considered within Panama, the presence of wALBb is unlikely to hinder control efforts, given that it is able to stably co-infect the mosquito host with minimal fitness costs^70^.

Until recently, it was widely accepted that *Ae*. *aegypti* does not habour natural infections of *Wolbachia*, although infection has since been reported in mosquitoes from Jacksonville and Houston, USA^1,32^. We found one individual of *Ae*. *aegypti* infected with *Wolbachia* in Veracruz, Panama, suggesting that although uncommon, it does occur and further screening would possibly yield further positives. A full scale assessment is required within Panama, with future work focused on understanding of the prevalence of natural *Wolbachia* infections acquired during the adult life stage and any potential barriers to mosquito dispersal, since these can impact on the success of *Wolbachia* spread^66,71^.

### Limitations of the study

There are potentially finer scale interspecific differences between *Ae*. *aegypti* and *Ae*. *albopictus* that are difficult to assess without controlling for the high degree of intraspecific bacterial variability we have observed within these mosquitoes. A better understanding of determinant factors including temporal changes in relation to the bacterial composition of habitat water is required to account for variability in individuals from the same oviposition site. Furthermore, there may be inter-specific differences acquired during the adult life stage, which were not the focus of this study^44^. Since there was limited information on *Aedes* distributions across Panama, we sampled mosquitoes systematically across the entire country in order to decipher both the competition landscape and bacterial associations under different habitat conditions. We found few sites of co-existence, highlighting biological competition as a potentially important determinant of species distributions. However, this could have limited our ability to decipher inter-species differences. Future studies may focus on areas of *Aedes* co-existence at the landscape and microhabitat levels, including within the Azuero Peninsula, Panama City or Colon. The resolution of the data depends on variability at the 16S rRNA region, primer affinity, and composition of the bacterial database^72^. Therefore, the exploration of *Aedes* bacterial communities with multiple genomic markers and culture dependent approaches with different media has the potential to increase the number or relative abundance of discoverable genera.

## Conclusion

Resident bacteria are likely to impact on mosquito host fitness, influence the outcome of biological competition and impact on vector competence, yet research efforts to gain insight into the dynamics and diversity of *Aedes* associated bacteria have been limited so far. Our study provides the basis for understanding the bacterial associations of *Aedes* mosquitoes across Panama, while highlighting the many avenues that remain to be explored. An understanding of the biological and ecological factors influencing bacterial associations will be paramount to resolve the consequences of the microbiota for mosquito-borne disease.

## Materials and Methods

### Mosquito collection and sample preparation

All *Aedes* mosquitoes were collected as larvae through the active surveillance of natural breeding sites around settlements across 37 locations and 59 oviposition sites in Panama (Fig. 1, Table S1). At the time of collection, the water temperature and pH were taken three times and averaged to account for variability in the readings. Additional details about our sampling protocol can be obtained from Eskildsen *et al*.^38^. Mosquitoes from each collection site were brought to the laboratory and reared to adulthood under standardized conditions (e.g., LD 12:12 hours, 85% relative humidity, and 30 °C, which are similar to those encountered throughout natural sites in Panama) in separate plastic glasses and the habitat water. On reaching fourth instar, larvae were placed on blotting paper and rinsed three times with distilled water to remove any surface bacteria. Larvae were stored dry in separate Eppendorf tubes or in 70% ethanol. Each emergent adult was placed at -20 °C for 20 minutes to induce death before transfer to a sterilised Eppendorf tube. Both larvae and adults were identified to species level using the morphological key of Rueda *et al*.^73^ and stored at −20 °C until DNA was extracted.

### DNA extraction, 16S rRNA gene library and sequencing

To assess whether pooling mosquitoes could capture the same level of bacterial diversity as processing individual mosquitoes, preliminary sequence data was captured for 7 pools of six *Ae*. *aegypti* and the component individual mosquitoes of those pools using the methods and computational processing described below. To analyse the whole-body microbial community of *Ae*. *aegypti* and *Ae*. *albopictus*, the DNA of 960 mosquitoes (470 *Ae*. *albopictus* and 490 *Ae*. *aegypti*) were extracted individually using a Biosprint® 96 DNA Blood kit (Qiagen). DNA pools were made by combining 2 µl of DNA extract from each of six individual mosquitoes from the same oviposition site (creating 75 sample pools of *Ae*. *albopictus* and 79 pools of *Ae*. *aegypti*). In addition, 20 *Ae*. *albopictus* and 16 *Ae*. *aegypti*, originating from oviposition sites with few mosquitoes, were processed individually. Pooled or individual DNA was then used to amplify the V4 region of the 16S rRNA^74^ locus in triplicate 12.5 μl reactions using a two-step PCR protocol. PCR 1 included 5 µl of 2X Maxima HotStart PCR Master Mix (Thermo), 0.2 µl of each primer (which included an Illumina sequencing primer on the 5′ end (10 mM)), and 1 µl of DNA. Cycling conditions had an initial denaturation step of 3 min at 94 °C proceeding 25 cycles of 94 °C for 45 sec, 50 °C for 60 sec, and 72 °C for 90 sec, followed by 10 min at 72 °C extension. The resulting triplicate PCR products were pooled and 1 µl used as template for a second PCR of six cycles to add on unique barcodes and Illumina sequencing adaptors. Resulting reactions were cleaned and normalized using PCR Normalization plates (Charm Biotech), pooled into a single species library and concentrated using Kapa magnetic beads. DNA concentration was verified with the Qubit HS assay (Invitrogen) and the quality checked with a Bioanalyzer dsDNA High Sensitivity assay. Libraries were sequenced on an Illumina MiSeq on a 2 × 250 paired end run.

### Data Analysis of 16S Metadata

The majority of analysis of sequences reads was performed using the Quantitative Insights Into Microbial Ecology (QIIME) software package versions 1.9.1 and 2.0^75^ using the forward reads of the V4 hypervariable region. The reverse reads were not used due to their reduced data quality, but results based on their analysis are provided for comparison as supplementary information. Quality control was performed with the Dada2 pipeline with forward sequences trimmed to 230 base pairs after the visual inspection of base quality score plots. The identities of OTU’s were designated using a Naive Bayes classifier trained on the Greengenes 99% sequence similarity database v13.8, where sequences were trimmed to 251 base pairs of the sequenced 16S regions bound by the 515F and 806R primer pair. Low abundance OTU’s (0.005%) were filtered from the resulting OTU table, recommended to remove potential sequencing errors^76^.

Informed by rarefaction curves, the feature table was standardised to a sequencing depth of 1000 before alpha and beta diversity values were calculated using the diversity core-metrics function in Qiime2^75^. Significance between the alpha diversity of metadata groups was determined using a non-parametric Mann-Whitney U test for two group comparisons in the R package Stats^77^. Significance differences between the beta diversity of metadata groups were calculated with PERMANOVA on both Bray-Curtis and unweighted UNIFRAC distance matrixes, as a widely applied and distribution-free test. To visualise dissimilarity between beta-diversity distance matrixes, Principle Coordinates Analysis (PCoA) plots were generated using unweighted UNIFRAC distances in the R program Phyloseq^78^. Heat maps were generated in Qiime 1 to visualise the profile of the microbial community with increasing values of water pH and temperature. To determine whether *Aedes* species could be accurately classified according to their microbial profile, a Random Forests regression model was run using the supervised learning classifier in Qiime2 with our microbial OTU data as a training dataset. Analysis was performed in the R package indicspecies^79^ to identify the bacterial OTU’s significantly indicative of the different *Aedes* species.

### Wolbachia PCR

Individual mosquitoes within pools positive for 16S *Wolbachia* were tested for the presence of the *Wolbachia* surface protein gene (*wsp*) in a PCR reaction using Maxima Hot Start Master Mix (Thermo Fisher Scientific) under the guidelines and PCR cycling conditions provided in Baldo *et al*.^80^. Positive PCR products were cut from an agarose gel, digested with GELase and purified with Serapure beads before sequencing on an ABI 3500XL Sanger Sequencer using BigDye Terminator chemistry. Resulting *Wolbachia wsp* sequences were edited and aligned in Geneious v11.0.5, assigned allele numbers and identified to strain using the *Wolbachia wsp* database (https://pubmlst.org/wolbachia). Data is available in GenBank (Accession numbers MH392327-MH392336).

## Supplementary information

Table S1

Table S2

Table S3

Table S4

Table S5

Table S6

Table S7

Table S8

Supplementary Information

## Data Availability

The 16S amplicon sequence datasets generated and analysed during the current study are available in the Genbank repository (BioProject PRJNA523634). *Wolbachia wsp* sequences are also available (Accession numbers MH392327-MH392336).
