# Supplementary material for: Dynamics and diversity of bacteria associated with the disease vectors Aedes aegypti and Aedes albopictus
Source: Sci Rep. 2019 Aug 21;9:12160. doi: 10.1038/s41598-019-48414-8 (PMC6704126; doi:10.1038/s41598-019-48414-8)
Supplement: Supplementary file 9 — Supplementary Information [file 41598_2019_48414_MOESM9_ESM.pdf]

Dynamics and diversity of bacteria associated with the disease vectors *Aedes aegypti* and *Aedes albopictus.*

Kelly L. Bennett [1,*], Carmelo Gómez-Martínez [1,2], Yamileth Chin [1], Kristin Saltonstall [1], W. Owen McMillan [1], Jose R. Rovira [1,2], & Jose R. Loaiza [1,2,3.*]

[1] Smithsonian Tropical Research Institute, Apartado 0843-03092, Balboa, Ancon, República de Panamá.

[2] Instituto de Investigaciones Científicas y Servicios de Alta Tecnología, Ciudad del Saber, Apartado 0843-01103, Panamá, República de Panamá.

[3] Programa Centroamericano de Maestría en Entomología, Universidad de Panamá, República de Panamá.

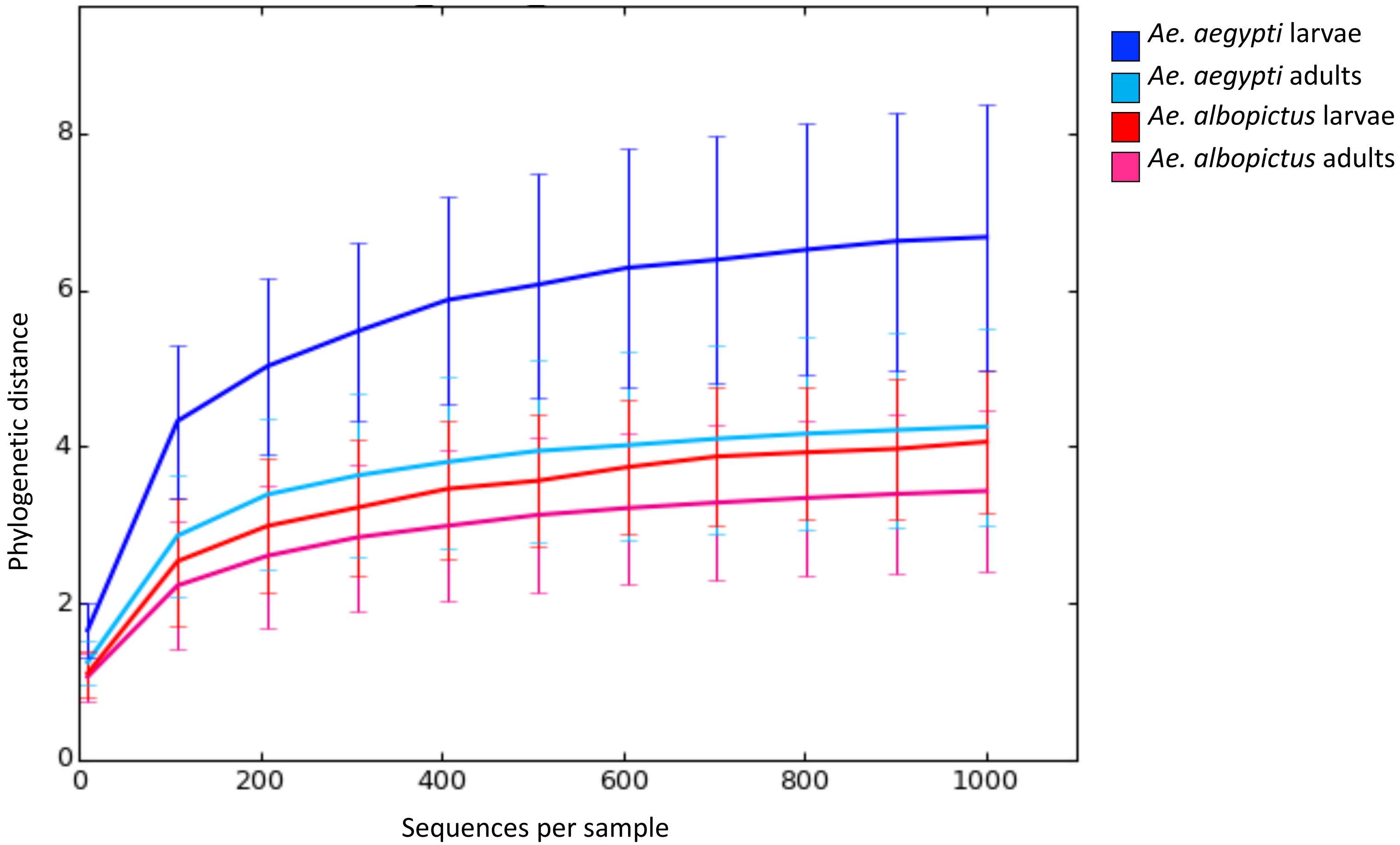

Figure S1. Rarefaction curves of alpha diversity of the bacterial communities found in larvae and adults of *Ae. aegypti* and *Ae. albopictus* collected from human inhabited environments across Panama as distinguished by life cycle stage. Mean Faith's phylogenetic diversity (±SE) of each sample group is shown across different sequencing depths

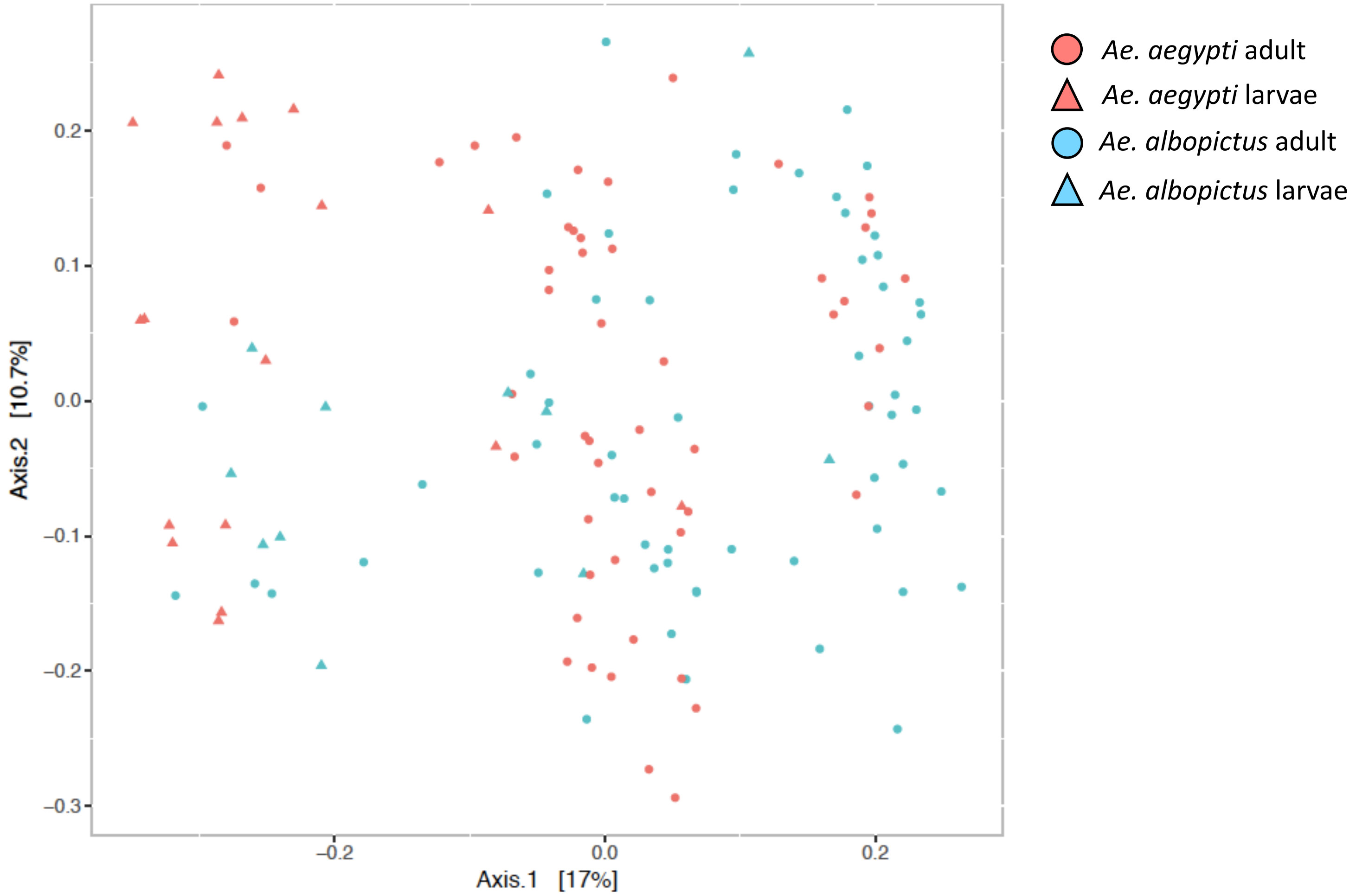

Figure S2. Principle Coordinates Analysis (PCoA) of unweighted UNIFRAC distances showing species differences in the microbial community coloured by mosquito species and life stage.

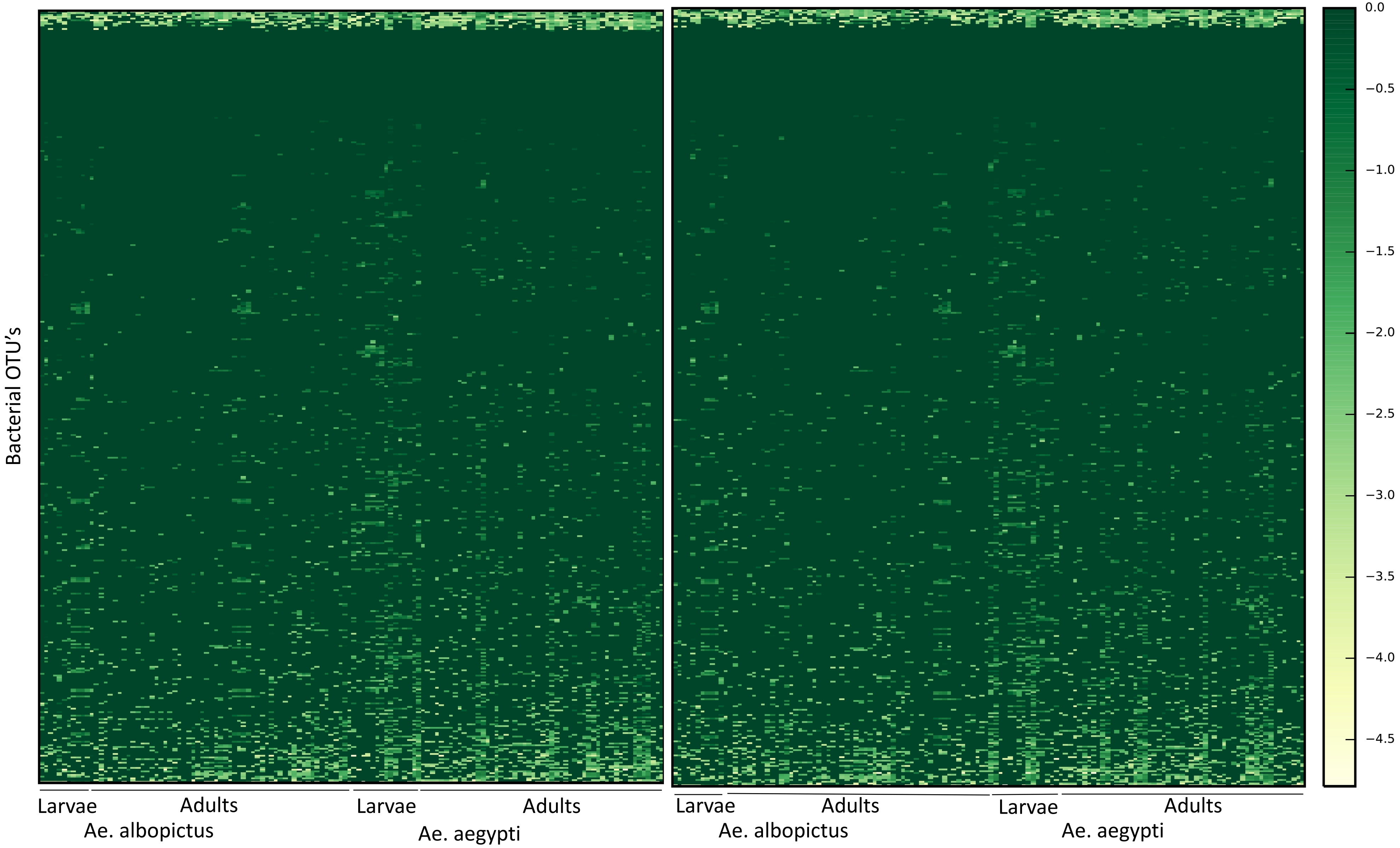

Figure S3. The relative abundance of bacterial OTU's with increasing values of water pH (left panel) and water temperature (right panel) of oviposition sites from which adult and immature *Ae. albopictus* and *Ae. aegypti* were collected.

Supplementary results based on the analysis of reverse reads

Composition and structure of *Aedes*-associated bacterial communities

In total, 7,309,396 sequence reads of bacterial 16S rRNA gene amplicons were generated from DNA pools and individually processed mosquitoes representing all samples of immature and adult *Ae. aegypti* and *Ae. albopictus*. The mean number of reads per pool or individual sample was 40,834 reads. Due to a reduction in the quality of reads, the reverse sequence data was trimmed at 120 base pairs (Figure S4). After quality filtering, 5,922,570 sequences were retained from 177 samples, including 75 pools (21.3 % larvae) and 20 individual adult *Ae. aegypti* and 62 pools (22.6 % larvae) and 20 individual adult *Ae. albopictus*. After rarefaction, these comprised 67 pools and 12 individuals of *Ae. aegypti* and 54 pools and 18 individuals of *Ae. albopictus* with 573 unique OTU's, averaged at 35 OTU's per individual or mosquito pool.

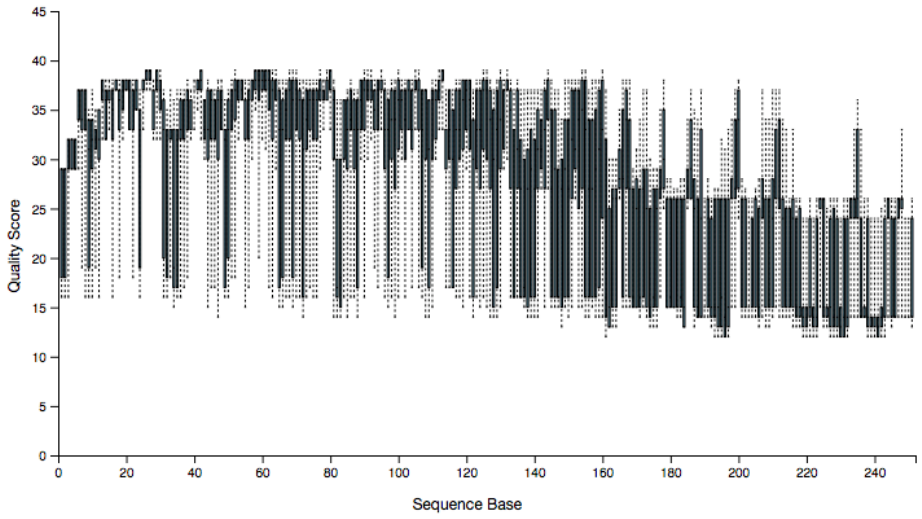

Figure S4. Bair pair quality plot of reverse reads showing that quality is reduced consistently to below a score of 20 after 120 base pairs.

In total, the bacterial communities analysed composed 16 phyla, 33 classes, 54 orders, 73 families and 74 genera of bacteria. Due to the reduced quality and length of the reverse reads, a larger proportion of bacterial OTU's were unidentified compared to the forward reads with 4 % and 2 % unassigned bacterial OTU's compared to 0.02 % and 0.00 % for *Ae. aegypti* and *Ae. albopictus* respectively. However, the dominant bacterial Phyla remained as *Proteobacteria,* which totalled 89 % of the identified OTU's for both mosquito species, in addition to *Bacteroidetes, Actinobacteria* and *Firmicutes* which were found at lower relative abundances above 1%. The composition of bacterial Phyla (Figure S5a) and Classes (Figure S5b) for each species and life stage are summarised here.

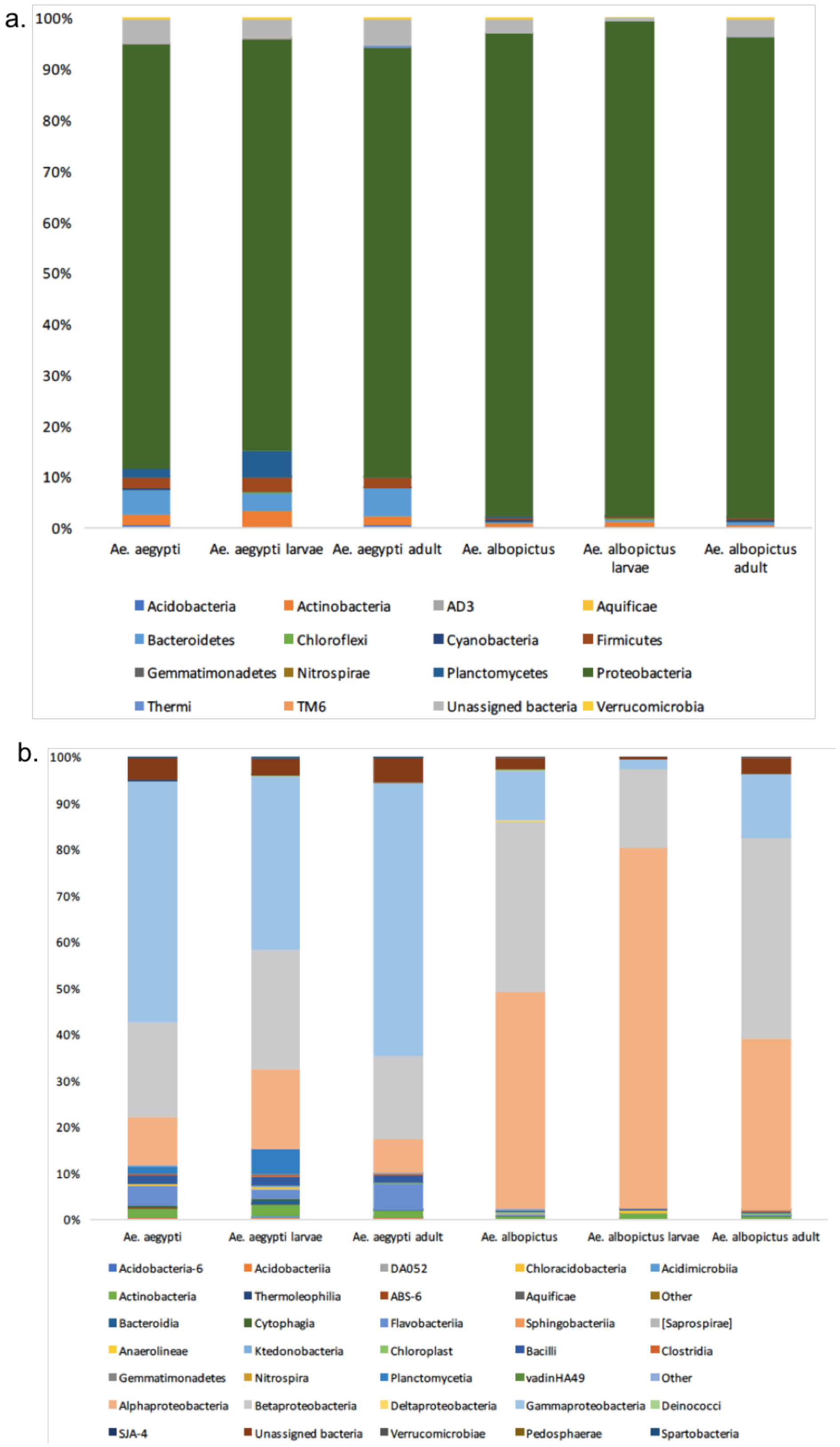

Figure S5. Bar plot of the relative abundances of a. the bacterial phyla and b. the bacterial classes within *Ae. aegypti* and *Ae. albopictus* summarized across all samples and compared to the bacterial profiles of the adults and larvae for each species.

Aedes intra- and inter-species bacterial diversity

Intraspecific variability within the bacterial communities of both *Aedes* species was high, with average Bray-Curtis distances of 0.80 and 0.83 for *Ae. aegypti* and *Ae. albopictus* respectively. *Flavobacteriales* were highly variable among pools of individuals, and dominated the bacterial community composition of a small number of samples from Los Santos province including three *Ae. aegypti* and one *Ae. albopictus* (Figure S6).

The bacterial diversity of larvae was higher and had a significantly different microbial community than emergent adults in *Ae. aegypti* (PERMANOVA of unweighted UNIFRAC distances, pseudo-F = 7.50, $P < 0.01$), but not *Ae. albopictus* (PERMANOVA of unweighted UNIFRAC distances, pseudo-F = 1.82, $P > 0.05$) (Table 1). This is likely due to a reduction in the number of identified OTU's overall for *Ae. aegypti* due to the lower quality and therefore shorter length of the reverse sequence data (Figure S4).

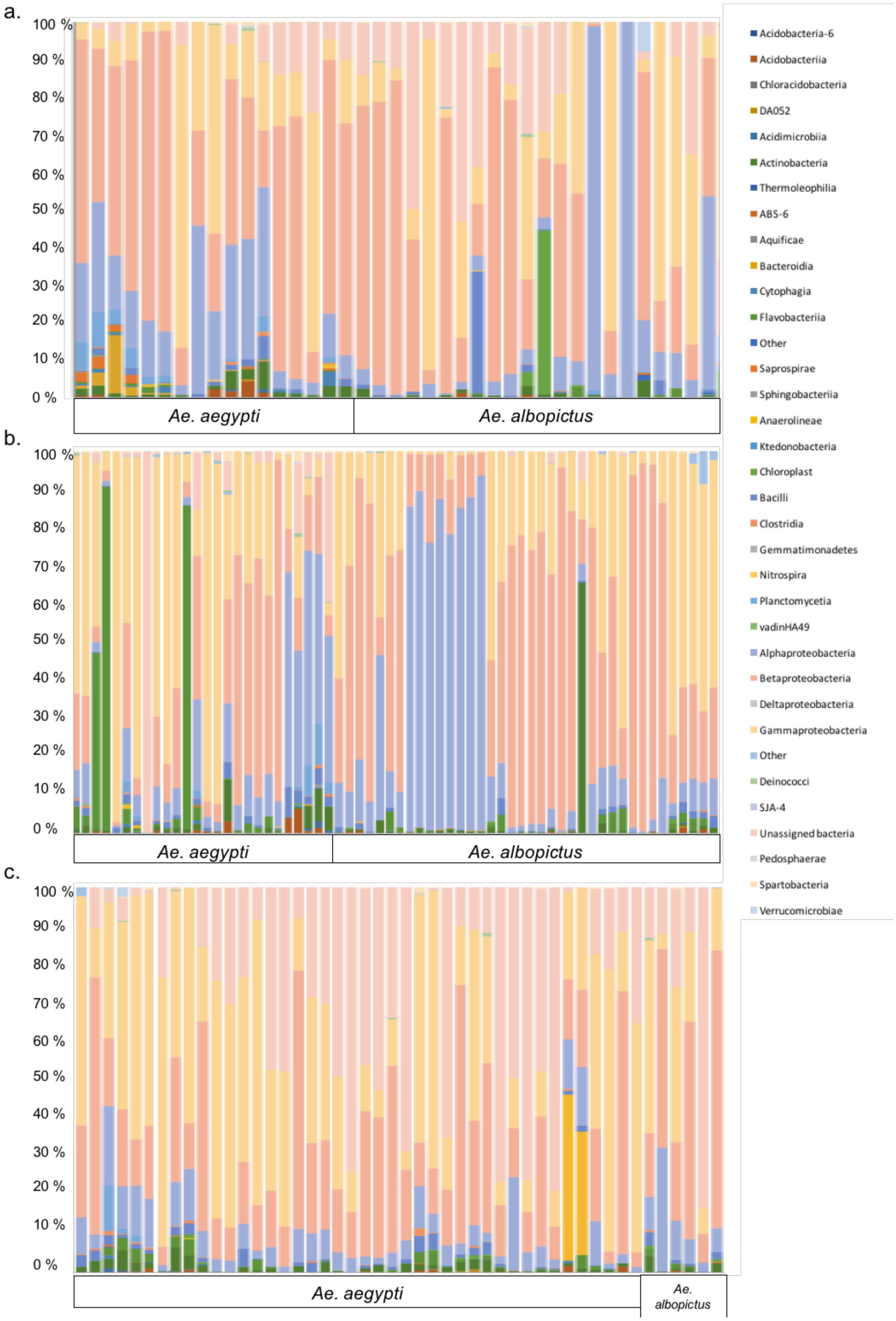

Figure S6. Bar plot to show the proportion of different bacterial classes within each mosquito or pool of mosquitoes tested from a. Western Panama including Bocas del Toro, Chiriquí, Herrera and Veraguas; b. Los Santos and c. Eastern Panama including Colón, Coclé, Darién and the province of Panamá.

Table 1. Mean number of operational taxonomic units (OTU's), Shannon's Diversity values (Shannon's D), Faith Phylogenetic diversity (Faith PD) and Evenness Index for each *Aedes* species in Panama.

		No. OTUs	Shannon's Diversity	Faith's PD	Evenness
Ae. aegypti		43.09	3.70	7.38	0.72
	Larvae	73.80	4.49	8.85	0.73
	Adults	35.89	3.51	7.03	0.71
Ae. albopictus		26.28	3.17	6.16	0.70
	Larvae	35.85	3.30	6.19	0.65
	Adults	24.17	3.14	6.16	0.71

Ae. aegypti and *Ae. albopictus* shared 52.9 % of bacterial OTU's, with a similar extent shared between species on comparison of only adults while a lesser extent was shared on comparisons of larvae (49.9 % and 33.8 % of OTU's, respectively). Despite species sharing a considerable proportion of bacteria, we found several rare taxa unique to *Ae. aegypti*, reflected in a higher bacterial diversity within larvae (Mann-Whitney U of Faith's PD, W = 2691 P < 0.01) and adults (Mann-Whitney U of Faith's PD, W = 131, P < 0.01) of this species when compared to *Ae. albopictus*. Furthermore, random forest analysis could successfully assign *Ae. aegypti* to the correct species class with a high accuracy of 0.94 and *Ae. albopictus* with a lower accuracy of 0.64. These estimates were similarly high on analysis of adults (~0.75 for both species) and larvae only (1 for both species). Analysis of the reverse reads with indicator species analysis identified a lower number of OTU's characteristic of *Ae. aegypti* (87) and *Ae. albopictus* (22) than the forward reads.

Larval habitat features and bacterial composition

We observed no consistent differences between both the bacterial community of larvae and adult *Aedes* species due to larval habitats features including geographic distribution, type of container material and associated environmental variables of the water. There were few statistically significant comparisons based on PERMANOVA between oviposition sites having different container materials for adults only (Table 2). Differences were observed between the region of Panama and Bocas del Toro/Los Santos for adult *Ae. aegypti* as well as between Los Santos and Chiriquí/Panamá for *Ae. albopictus* adults, but not for larvae (Table 3). Similar to analysis based on forward reads, the water temperature and pH of the larval habitat did not strongly influence the microbiome acquired by the mosquito host, with no clear change in the microbial community with increasing values of either variable in both species.

Table 2. PERMANOVA test of differences between the microbial community of mosquitoes from oviposition sites of different materials. The results of significant comparisons are highlighted in bold.

Species		Group comparisons			PERMANOVA based on Bray-Curtis distances			PERMANOVA based on Unifrac distances		
		Group 1	Group 2	Sample size	pseudo-F	p-value	q-value	pseudo-F	p-value	q-value
Ae. aegypti	Adults	**plastic**	**rubber**	**57**	**3.055**	**0.001**	**0.003**	**3.561**	**0.010**	**0.030**
		plastic	wood	35	1.921	0.016	0.024	1.756	0.139	0.209
		rubber	wood	30	0.839	0.663	0.663	0.827	0.486	0.486
	Larvae	metal	plastic	10	3.574	0.006	0.018	2.288	0.031	0.093
		metal	rubber	11	2.717	0.017	0.026	1.488	0.179	0.269
		plastic	rubber	13	1.305	0.244	0.244	0.881	0.430	0.430
Ae. albopictus	Adults	ceramic	natural	13	1.409	0.215	0.258	1.401	0.173	0.208
		ceramic	plastic	33	4.490	0.001	0.002	2.330	0.048	0.072
		**ceramic**	**rubber**	**35**	**2.588**	**0.001**	**0.002**	**13.985**	**0.001**	**0.003**
		natural	plastic	28	1.009	0.427	0.427	1.137	0.359	0.359
		**natural**	**rubber**	**30**	**1.144**	**0.200**	**0.258**	**6.022**	**0.002**	**0.004**
		**plastic**	**rubber**	**50**	**4.398**	**0.001**	**0.002**	**15.467**	**0.001**	**0.003**
	Larvae	ceramic	rubber	9	6.051	0.009	0.009	1.989	0.113	0.113

Table 3. PERMANOVA test of differences between the microbial community of mosquitoes in the different provinces of Panama. The results of significant comparisons are highlighted in bold.

Species	Lifestage				PERMANOVA based on Bray-Curtis Distances			PERMANOVA based on UNIFRAC distances		
		Group 1	Group 2	Sample size	pseudo-F	p-value	q-value	pseudo-F	p-value	q-value
Ae. aegypti	Adults	Bocas del Toro	Chiriquí	7	1.519	0.147	0.523	1.920	0.189	0.472
		Bocas del Toro	Coclé	6	1.314	0.336	0.523	1.006	0.656	0.758
		Bocas del Toro	Colon	8	1.179	0.235	0.523	2.304	0.057	0.319
		Bocas del Toro	Darién	8	1.183	0.316	0.523	1.929	0.172	0.472
		Bocas del Toro	Herrera	7	2.261	0.037	0.235	1.997	0.176	0.472
		Bocas del Toro	Los Santos	25	1.061	0.355	0.523	0.586	0.677	0.758
		**Bocas del Toro**	**Panamá**	**30**	**2.776**	**0.001**	**0.014**	**9.830**	**0.001**	**0.014**
		Chiriquí	Coclé	3	0.763	1.000	1.000	0.746	1.000	1.000
		Chiriquí	Colon	5	0.781	0.609	0.656	1.231	0.406	0.541
		Chiriquí	Darién	5	1.479	0.313	0.523	1.125	0.299	0.506
		Chiriquí	Herrera	4	0.780	1.000	1.000	1.466	0.325	0.506
		Chiriquí	Los Santos	22	1.478	0.100	0.467	1.548	0.219	0.472

		Chiriquí	Panamá	27	0.955	0.504	0.604	0.418	0.903	0.936
		Coclé	Colón	4	1.186	0.518	0.604	0.682	0.731	0.787
		Coclé	Darién	4	1.764	0.269	0.523	0.654	0.506	0.616
		Coclé	Herrera	3	3.670	0.351	0.523	2.506	0.313	0.506
		Coclé	Los Santos	21	1.156	0.433	0.604	0.985	0.324	0.506
		Coclé	Panamá	26	1.069	0.350	0.523	0.922	0.382	0.535
		Colón	Darién	6	0.979	0.476	0.604	1.058	0.473	0.602
		Colón	Herrera	5	2.236	0.220	0.523	1.608	0.194	0.472
		Colón	Los Santos	23	0.912	0.500	0.604	1.903	0.149	0.472
		Colón	Panamá	28	1.897	0.024	0.224	3.128	0.022	0.154
		Darién	Herrera	5	2.735	0.193	0.523	1.885	0.209	0.472
		Darién	Los Santos	23	0.839	0.553	0.619	1.608	0.236	0.472
		Darién	Panamá	28	1.290	0.191	0.523	5.682	0.007	0.065
		Herrera	Los Santos	22	1.926	0.042	0.235	1.720	0.115	0.472
		Herrera	Panamá	27	1.338	0.152	0.523	1.051	0.355	0.523
		**Los Santos**	**Panamá**	**45**	**4.780**	**0.001**	**0.014**	**11.961**	**0.001**	**0.014**
	Larvae	Bocas del Toro	Chiriquí	9	1.069	0.393	0.393	1.645	0.162	0.324
		Bocas del Toro	Colón	9	2.372	0.051	0.153	2.691	0.053	0.237
		Bocas del Toro	Los Santos	13	2.210	0.031	0.153	1.999	0.079	0.237
		Chiriquí	Colón	4	2.335	0.337	0.393	2.747	0.349	0.349
		Chiriquí	Los Santos	8	1.409	0.250	0.375	1.007	0.340	0.349
		Colón	Los Santos	8	1.478	0.232	0.375	1.676	0.256	0.349
Ae. albopictus	Adults	Chiriquí	Herrera	19	1.033	0.348	0.388	0.784	0.522	0.522
		**Chiriquí**	**Los Santos**	**45**	**3.722**	**0.001**	**0.003**	**14.482**	**0.001**	**0.003**
		Chiriquí	Panamá	25	1.281	0.164	0.251	2.182	0.042	0.063
		Herrera	Los Santos	32	1.017	0.388	0.388	2.658	0.034	0.063
		Herrera	Panamá	12	1.247	0.167	0.251	1.595	0.146	0.175
		**Los Santos**	**Panamá**	**38**	**3.839**	**0.001**	**0.003**	**18.233**	**0.001**	**0.003**
	larvae	Colón	Los Santos	7	5.312	0.053	0.159	1.003	0.503	0.641
		Colón	Panamá	3	6.040	0.343	0.343	1.122	0.641	0.641
		Los Santos	Panamá	6	3.080	0.320	0.343	1.811	0.153	0.459

Wolbachia **occurrence and distribution**

We found *Wolbachia* 16S rDNA positive samples in both *Aedes* species. This included 12 pools and four individuals of *Ae. albopictus* of both adults and larvae widespread across Panama and two adult *Ae. aegypti* from Los Santos and Panama province. The outcomes on the distribution of Wolbachia across Panama were not affected by changes in the quality of sequence reads because they were additionally supported through conventional PCR.

Conclusion

Although results based on the analysis of low-quality reverse reads largely agree with the findings and interpretations of our original submission, we lose resolution to make comparison at the intraspecific level within and between populations of *Ae. aegypti* and *Ae. albopictus* from across Panama. Therefore, we decided to exclude reverse reads from final analyses due to their lower quality.
